# Recent Advances in Mechanics of Materials

**DOI:** 10.3390/ma19122453

**Published:** 2026-06-08

**Authors:** Weidong Song, Yingying Xue

**Affiliations:** State Key Laboratory of Explosion Science and Safety Protection, Beijing Institute of Technology, Beijing 100081, China; xyy_0806@126.com

The mechanics of materials is a fundamental interdisciplinary field, underpinning innovations in aerospace, marine engineering, construction, and advanced manufacturing [1,2,3]. It focuses on elucidating the mechanical behaviors, damage mechanisms, and performance optimization of materials under various loading conditions, providing critical theoretical support for the design, fabrication, and servicing of high-performance structural components [4,5,6,7,8]. This Special Issue (SI), titled “Mechanics of Materials,” presents recent advances regarding the mechanical properties of advanced materials. This editorial synthesizes the key findings of eight representative papers and highlights their core contributions to the field.

Low-cycle fatigue (LCF) is a major failure mode for S420M steel, which is widely used in the load-bearing components of power plants, bridges, and wind farms [9]. Pre-strain, often introduced by accidental static overloads during service, is typically overlooked in traditional LCF studies [10]. Given that S420M steel does not exhibit cyclic stabilization under strain-controlled LCF, ignoring pre-strain may lead to inaccurate fatigue life predictions and potential safety risks [11,12]. Mroziński et al. [13] demonstrated that pre-strain reduces hysteresis loop energy across all strain amplitudes and slightly improves the fatigue life of S420M steel, with statistically consistent energy fatigue characteristics observed between pre-strained and as-received specimens. Theoretically, this study fills a gap by clarifying how pre-strain influences energy dissipation and fatigue durability and confirms the applicability of existing analytical models to pre-strained components.

Lattice structures offer advantages such as lightness, a high specific strength, and excellent energy absorption capacity, making them valuable for lightweight design and protection in marine, aerospace, automotive, and medical applications [14,15]. Zhang et al. [16] investigated the underwater explosion resistance of aluminum alloy octet-truss lattice sandwich structures using shock tube experiments, LS-DYNA simulations, and 3D-DIC deformation measurements. They analyzed dynamic deformation mechanisms and energy absorption characteristics, quantifying the effects of lattice relative density and target plate thickness on protective performance. Additionally, a novel I-WP-X lattice structure was designed by fusing an X-shaped plate with an I-WP surface. Ti6Al4V lattice specimens fabricated via selective laser melting (SLM) were tested under compression and simulated using finite-element methods, demonstrating significant performance improvements over conventional designs [17].

Carbon-fiber-reinforced polymer (CFRP) laminates are widely employed in aerospace, marine, and defense fields due to their high specific strength and corrosion resistance [18]. In engineering applications, CFRP laminates often suffer from single waterjet impacts [19]. Hou et al. [20] elucidated the damage mechanisms of CFRP laminates affected by single waterjet impacts from different directions, resolving a long-standing debate regarding the influence of impact orientation on damage. The established damage model fills a quantitative prediction gap for CFRP waterjet impact damage, providing reliable technical support for the design and service safety of CFRP components in aerospace, ocean engineering marine engineering, and other fields [21]. This work also enriches CFRP impact resistance research, laying a foundation for structural optimization and material selection.

Amorphous silica is a versatile material with broad application prospects in regard to aerospace, semiconductors, biomedicine, and construction due to its unique mechanical, optical, and thermal properties [22]. Its service life is limited by fracturing, a critical failure mechanism, and the fracture surfaces of amorphous silica exhibit distinct fractal characteristics [23]. Morin et al. [24] employed semi-empirical quantum mechanical simulations combined with a continuous random network model and reaction coordinate method to investigate the fractal fracture process of amorphous silica. This study provides a reference for quantum simulations of fractal fracturing and establishes a quantum mechanical model that links atomic-scale bond reconfiguration with macroscopic fractal fracture characteristics.

Graphene oxide (GO), a derivative of graphene, has attracted significant tribological interest due to its unique layered morphology, high specific surface area, and abundant oxygen-containing functional groups [25]. It is commonly used in the automotive, aerospace, biomedical, and manufacturing industries to improve friction and wear performance in mechanical systems [26]. Wakchaure et al. [27] systematically reviewed recent advances in the tribological performance of GO and its composites. Their review addresses the lack of a comprehensive synthesis of the latest advances in GO tribology, clarifying the enhancement mechanisms and key influencing factors. It provides a theoretical reference for future research on GO-based tribological materials and guides the rational design and modification of GO composites to overcome inherent limitations [28,29].

Two-component polyurethane (2C PU) adhesives are widely applied in the construction, automotive, and manufacturing industries due to their excellent bonding strength, flexibility, and chemical resistance [30]. Chalk (calcium carbonate) is a traditional inorganic filler used to reduce production costs and adjust rheological properties [31]. However, widespread chalk usage increases resource consumption, while fly ash, a solid waste product of coal combustion, poses environmental challenges due to massive accumulation, and there is a lack of high-value fly ash utilization strategies [32]. Although recent studies have explored using fly ash as a filler in PU adhesives, systematic research on the effects of replacing chalk with fly ash on the physicochemical and mechanical properties of 2C PU adhesives is lacking. The optimal substitution ratio and underlying mechanisms remain unclear. Pęczek et al. [33] clarified the impact of substituting chalk with fly ash on the properties of 2C PU adhesives, determining the optimal substitution ratio and providing a theoretical basis for formulating sustainable PU adhesives. This work enables high-value utilization of fly ash and reduces environmental pollution and production costs, aligning with circular economy principles.

European oak and Norway spruce clear wood are two of the most important commercial wood species in Europe and widely applied in structural engineering, furniture manufacturing, and low-carbon construction because of their renewability, high specific strength, and good processability [34]. As a defect-free material, clear wood is the core component of high-performance wood products, and its mechanical properties directly determine the safety, durability, and service life of end products, which are also the critical factors for the rational design and application of timber structures. Gambarelli et al. [35] filled a gap in the systematic experimental research on the mechanical properties of these two species, particularly regarding their complete stress–strain response and orthotropic behavior. This study clarifies the differences in mechanical properties between European oak and Norway spruce and reveals the influence of key factors such as moisture content and annual ring width, providing accurate and standardized data for timber structure design and safety evaluation.
